# Radiomic Analysis to Predict Outcome in Recurrent Glioblastoma Based on Multi-Center MR Imaging From the Prospective DIRECTOR Trial

**DOI:** 10.3389/fonc.2021.636672

**Published:** 2021-04-14

**Authors:** Alex Vils, Marta Bogowicz, Stephanie Tanadini-Lang, Diem Vuong, Natalia Saltybaeva, Johannes Kraft, Hans-Georg Wirsching, Dorothee Gramatzki, Wolfgang Wick, Elisabeth Rushing, Guido Reifenberger, Matthias Guckenberger, Michael Weller, Nicolaus Andratschke

**Affiliations:** ^1^Department of Radiation Oncology, University Hospital Zurich, Zurich, Switzerland; ^2^Department of Neurology, University Hospital Zurich, Zurich, Switzerland; ^3^Neurology Clinic, University Heidelberg Medical School, Heidelberg, Germany; ^4^Department of Neuropathology, University Hospital Zurich, Zurich, Switzerland; ^5^Department of Neuropathology, Heinrich Heine University Düsseldorf, Düsseldorf, Germany

**Keywords:** radiomics, recurrent glioblastoma, *MGMT* status, DIRECTOR trial, linear intensity interpolation

## Abstract

**Background:**

Based on promising results from radiomic approaches to predict *O^6^-methylguanine DNA methyltransferase promoter methylation status* (*MGMT* status) and clinical outcome in patients with newly diagnosed glioblastoma, the current study aimed to evaluate radiomics in recurrent glioblastoma patients.

**Methods:**

Pre-treatment MR-imaging data of 69 patients enrolled into the DIRECTOR trial in recurrent glioblastoma served as a training cohort, and 49 independent patients formed an external validation cohort. Contrast-enhancing tumor and peritumoral volumes were segmented on MR images. 180 radiomic features were extracted after application of two MR intensity normalization techniques: fixed number of bins and linear rescaling. Radiomic feature selection was performed *via* principal component analysis, and multivariable models were trained to predict *MGMT* status, progression-free survival from first salvage therapy, referred to herein as PFS_2_, and overall survival (OS). The prognostic power of models was quantified with concordance index (CI) for survival data and area under receiver operating characteristic curve (AUC) for the *MGMT* status.

**Results:**

We established and validated a radiomic model to predict *MGMT* status using linear intensity interpolation and considering features extracted from gadolinium-enhanced T1-weighted MRI (training AUC = 0.670, validation AUC = 0.673). Additionally, models predicting PFS_2_ and OS were found for the training cohort but were not confirmed in our validation cohort.

**Conclusions:**

A radiomic model for prediction of *MGMT* promoter methylation status from tumor texture features in patients with recurrent glioblastoma was successfully established, providing a non-invasive approach to anticipate patient’s response to chemotherapy if biopsy cannot be performed. The radiomic approach to predict PFS_2_ and OS failed.

## Introduction

Glioblastoma is the most commonly occurring and aggressive malignant brain tumor in adults (1) and is classified by the World Health Organization as astrocytoma grade 4 (2). Patients enrolled in clinical trials show a dismal outcome with a median overall survival (OS) of 14.6 to 16.8 months and a 2 year survival rate of 27.2 to 33.9% (3–5). For newly diagnosed glioblastoma, the standard of care consists of gross total tumor resection when feasible followed by involved field radiotherapy as well as concomitant and sequential chemotherapy with the alkylating agent temozolomide. In contrast, for recurrent disease, optimal salvage therapy has not been defined with data lacking on predictive factors and superiority of the various treatment options (6). Glioblastoma is a heterogeneous tumor entity with various prognostic and predictive factors, including clinical (patient age, Karnofsky performance status) and molecular characteristics (O^6^-methylguanine DNA methyltransferase promoter methylation status, *MGMT* status) (7, 8) affecting survival and treatment response. *MGMT* status is a well-established biomarker for newly diagnosed and recurrent glioblastoma and is predictive for both overall survival and treatment response to temozolomide (9). In a systematic review, temozolomide was found effective in recurrent glioblastoma (10) and possibly superior to nitriosurea-based chemotherapy (11, 12). The hypothesis of dose-dense or metronomic application being superior to the conventional schedule could not be confirmed in later randomized trials (9, 11). Yet, the DIRECTOR trial could establish the predictive role of *MGMT* status for response to temozolomide (9). Furthermore, the field of radiomics has introduced a large number of non-invasive medical imaging characteristics to describe specific phenotypic differences of tumors. Accordingly, several quantitative radiomic approaches have shown their potential to predict *MGMT* status and clinical outcome in patients with newly diagnosed glioblastoma (13–16). However, there are no reliable data on the value of quantitative radiomics for recurrent glioblastoma. This study was designed to evaluate the association of clinical outcome (progression-free survival, PFS_2_, and OS) and molecular characteristics (*MGMT* status) with radiomic features from tumoral and peritumoral tissue on gadolinium-enhanced T1-weighted MR images in glioblastoma patients at first progression.

## Materials and Methods

### Patient Population

MR imaging data of the DIRECTOR trial (9), a prospective randomized multicenter trial that compared two dose-intensified temozolomide regimens in recurrent glioblastoma either with or without repeat surgery, were retrospectively analyzed and represented the training cohort (N = 105). The DIRECTOR trial showed a similar outcome in both study arms. Furthermore, imaging data were examined from an independent validation cohort (N = 49) at the University Hospital of Zurich enrolled by the same inclusion criteria as in the DIRECTOR trial (**Table S1**). All patients underwent prior treatment with standard of care for newly diagnosed glioblastoma (gross total resection if feasible followed by involved field radiotherapy and concomitant and sequential temozolomide chemotherapy) and were monitored for disease status by MRI in 8-week-cycles. DIRECTOR patients were excluded by the following criteria: unavailable pre- and post-contrast T1-weighted MR imaging data at recurrence prior to second surgery (N = 30), slice thickness of imaging data larger than 6.6 mm (N = 1), tumor volume at recurrence smaller than 0.2 ml (N = 4), newly diagnosed tumor located in the spinal cord (N = 1). Finally, 69 patients remained in the training cohort. Patient and imaging characteristics are summarized in **Table 1** and inclusion and exclusion criteria in **Table S1**. PFS_2_ (in contrast to the time of diagnosis to first progression, PFS_1_) and OS as clinical and *MGMT* status as epigenetic characteristic(s) were available. PFS_2_ was defined as the duration from the date of first progression until further progression. OS was defined as the duration from the date of the first progression to the date of tumor-related death. According to available literature, *MGMT* status rarely changes in the course of disease (17); therefore, *MGMT* status was determined by tissue analysis either from newly diagnosed or recurrent tumor. In the training cohort at the time of data analysis (April 17, 2015), tumor progression was documented in 65 patients and tumor related death in 61 patients out of all 69 patients. All patients gave written informed consent, and the study was approved by the local ethics committees and designated authorities (KEK-ZH-Nr. 20140540, KEK-ZH-Nr. 2009-0135/1, KEK-ZH-Nr. 2015-0437).

**Table 1:** Clinical characteristics of studied patient cohorts (training and validation) and imaging protocol details.

Characteristic		Training cohort	Validation cohort
		N = 69	N = 49
Age at diagnosis	Median (y)	58	53
	Range (y)	37–77	38–77
Sex	Female	26 (38%)	12 (24%)
	Male	43 (62%)	37 (76%)
*MGMT* status	Methylated	28 (41%)	17 (52%)
	Unmethylated	41 (59%)	16 (48%)
	No data	0	16
Median survival	PFS_2_ (mo, range)	2.7 (0–63)	3.7 (1–31)
	OS (mo, range)	11.3 (2–63)	13.4 (2–84)
KPS at first progression	90-100	37 (54%)	29 (59.2%)
	70-80	23 (33%)	17 (34.7%)
	<70	9 (13%)	3 (6.1%)
Steroids at first progression	Yes	20 (35%)	12 (24.5%)
	No	38 (65%)	37 (75.5%)
	No data	11	0
Second surgery	Yes	42 (61%)	20 (41%)
	No	27 (39%)	29 (59%)
Median VOI	Tumoral (ml, range)	11.7 (0.23–121.3)	5.0 (0.39–48.9)
	Peritumoral (ml, range)	75.2 (7.35–204.5)	53.0 (22.2–203.8)
MR Scanner	GE Medical Systems	N = 6	N = 17
	Discovery MR750	0	2 (4.1%)
	Signa Excite	0	5 (10.2%)
	Signa HDxt	6 (8.7%)	10 (20.4%)
	Philips Healthcare	N = 24	N = 22
	Ingenia	0	12 (24.5%)
	Achieva	3 (4.4%)	4 (8.2%)
	Intera	21 (30.4%)	6 (12.2%)
	Siemens	N = 39	N = 10
	Aera	1 (1.4%)	0
	Avanto	6 (8.7%)	0
	Numaris 3D	1 (1.4%)	0
	Skyra	4 (5.8%)	8 (16.4%)
	Sonata	9 (13%)	1 (2%)
	Symphony	5 (7.3%)	0
	TrioTim	5 (7.3%)	0
	Verio	6 (8.7%)	1 (2%)
	No data	2 (2.9%)	0
Magnetic field strength	1.0 T	1 (1.4%)	0
	1.5 T	39 (63.8%)	25 (51%)
	3.0 T	24 (34.8%)	24 (49%)
Image parameters	Slice thickness (mm, range)	0.44–6.6	0.7–5.3
	In-plane resolution (mm, range)	0.36–1.20	0.38–0.99

MGMT status, O^6^-methylguanine-DNA methyltransferase promoter methylation status; PFS_2_, progression-free survival; OS, overall survival; KPS, Karnofsky performance status; VOI, volume of interest.

### Image Acquisition and Segmentation

**Figure 1:**
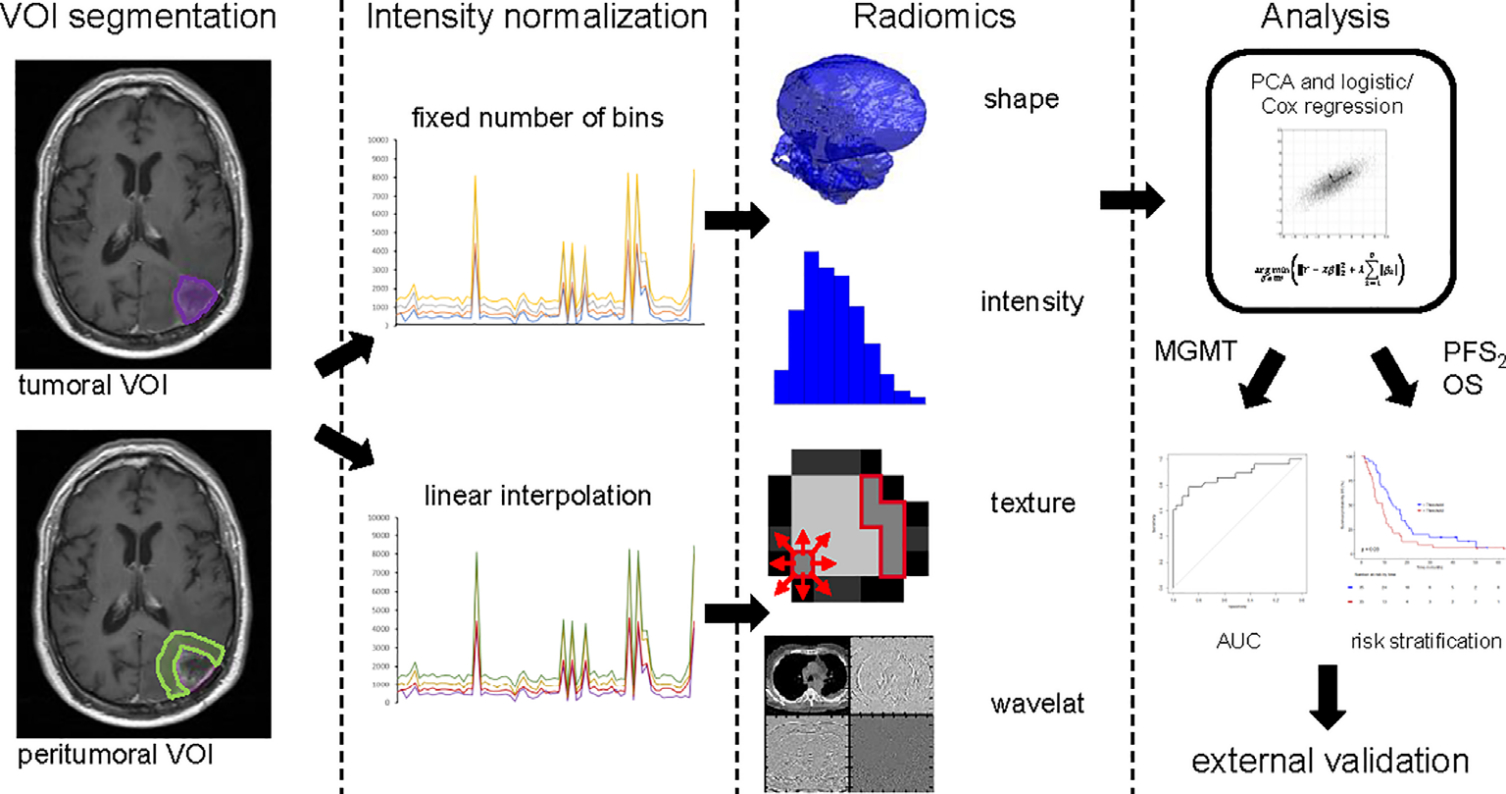
Image postprocessing and radiomics workflow. VOI Segmentation: Tumor contour (violet) and peritumoral area (light green) are shown on MRI imaging data. Intensity normalization: Either with fixed numbers of bins or linear interpolation. Radiomics: Features of three groups’ shape, intensity and texture were extracted with in-house developed software. Analysis: The statistical process of radiomic feature selection and risk stratification is shown up to validation on an external cohort. VOI, volume of interest; PCA, principal component analysis; *MGMT* status, O6-Methylguanin-DNA-Methyltransferase promoter methylation status; PFS_2_, progression free survival; OS, overall survival.

MRI data, acquired by either 1, 1.5, or 3 T systems, according to the protocols at each institution, were available. Technical data are shown in **Table 1**. The segmentation of the tumor volume was performed manually by medical doctors (AV, JK) on gadolinium contrast-enhanced T1-weighted MRIs using MIM VISTA (Version 6.7.9., MIM software Inc., Cleaveland, USA) and audited by a senior radiation oncologist with 10 years of experience after board certification (NA). Tumor volume of interest (VOI) included contrast-enhancing and cystic or necrotic areas. The resection cavity from first surgery was excluded if no sign of contrast enhancement was present. If blood residuals were seen along the border of the resection cavity, we subtracted hyperintense pre-contrast T1 volume from the post-contrast T1 volume. Segmentation of the peritumoral VOI was performed semi-automatically using an in-house developed MIM workflow. An isotropic 15-mm rim around the tumor volume was then generated. Non-brain tissue such as resection cavities, ventricle, subarachnoid space, and cranial bone were excluded manually by authors AV and JK and checked by author NA. Both tumor and peritumoral volumes of post-contrast T1-weighted images were considered for radiomic feature extraction (**Figure 1**).

### Image Postprocessing and Radiomic Feature Extraction

Prior to radiomic feature extraction, images were resized to cubic voxel size of 1 mm for shape analysis and to cubic voxel size of 3 mm for intensity and texture analysis using trilinear interpolation. The training cohort consisted of multicenter data with variable imaging protocols, thus prior to radiomic feature extraction, intensity normalization was performed. Currently, there are no consensus guidelines regarding a standard MRI intensity normalization technique for radiomic feature calculation. Therefore, we investigated two methods and compared prognostic power of MRI-based radiomics after use of these two normalized techniques (**Figure 1**). In the first normalization approach, the entire range of intensities was divided into a series of 32 bins (fixed bin number normalization). In the second approach, a linear intensity interpolation was used with two fixed tissues of reference: white matter of the contralateral brain tissue and vitreous body of one eye (Function showing the relation of the original to the transformed intensities, **Figure S1**). Additionally, texture features were extracted using fixed bin size of 50. Bin size was adjusted in the second method, so that the number of bins was similar to the number analyzed with the first method. Large variations in the number of bins between the methods would result in differential sensitivity to noise and the values of both normalization techniques would then not be comparable. Feature extraction and statistical analysis were done for both techniques.

Radiomic feature extraction was performed with the in-house developed software Z-Rad (18) written in Python programming language (v 2.7, Python Software Foundation, Delaware, USA). This software was benchmarked in the Image Biomarker Standardization Initiative (19) and allocates three-dimensional image analysis including all four feature extraction methods: shape, intensity, texture, and wavelet transformation. For our analysis, a total number of 180 features (shape N = 24, intensity N = 19, and texture N = 137, full list represented in the **Table S2**) per tumoral and peritumoral volume per patient were calculated. Intensity and texture analyses were performed, as mentioned, using 32 bins or linear rescaling with bin size of 50. All wavelet features were excluded due to the small volumes of the analyzed regions (**Figure 1**).

### Statistical Analysis

The methods applied were developed by our group and recently published (20). Briefly, this statistical method reduces feature space and correlates independent radiomic features with clinical endpoints to find prognostic or predictive biomarkers. The statistical analysis was performed in R (Version 3.5.3, The R Foundation, Vienna, Austria) (21). For both intensity normalization techniques and both volumes, tumoral and peritumoral, the statistical analysis was done separately. In total, per endpoint four models were trained using two intensity normalization techniques and two volumes of interest. To account for inter-features, correlation radiomic features were scaled and subsequently grouped according to the principal component analysis. The optimal number of retained principal components was determined using the Horn method (22). Each feature was assigned to the retained principal component to which the contribution was the greatest. Each of these principal components represented one group of correlated features (**Figure 1**).

#### Prediction of *MGMT* Promoter Methylation Status

Univariate logistic regression analysis was used to identify correlated radiomic features (p < 0.05) for *MGMT* status for both tumoral and peritumoral volumes. Only the most prognostic and significant (p < 0.05) feature per principal component feature group was included in further analysis. Feature prognostic value was quantified by area under receiver operating characteristic curve (AUC). The preselected features were enrolled in the multivariable logistic regression analysis, and the Akaike information criterion in the backward selection of variables was used to build the multivariable model. All results were verified in the independent external validation cohort. Additionally, an internal 5-fold-cross validation was performed (**Figure 1**).

#### Radiomics Prognostic Value of PFS_2_ and OS

Similar statistical methods were used to predict PFS_2_ and OS. Again, we analyzed tumoral and peritumoral volumes. Univariable Cox regression analysis was used to select the most prognostic feature (concordance index, CI) representing each principal component feature group. Only one feature per group was included in the multivariable analysis. Again, we used the Akaike information criterion in the backward selection of variables to build the final model, and all results were verified in an external validation cohort. Further, an internal 5-fold-cross validation was performed. Both training and validation cohorts were split into two prognostic groups based on the optimal threshold to generate survival curves and compared using log-rank test (p < 0.05). The threshold was defined as the median prediction value in the training cohort calculated from the final model using the prediction function in R (**Figure 1**).

#### Image Quality

Variations in image quality between different cohorts may also be critical for radiomic features and modeling. These variations may be identified by visual inspection of individual MRI datasets. However, such an approach is laborious, not sensitive to subtle variations between MR images (23) and subjective due to high inter-rater variability (24). In this work, the image quality of two cohorts was investigated by the semi-automatic approach using open-source tool MRQy. This tool is based on the HistoQC Python framework (25) and allows for automatic foreground detection for any MR image and extraction of imaging-specific metadata and quality measures. The major components of the MRQy tool and all the measures extracted by MRQy were described by Sadri et al. (26). We divided all investigated metrics in four groups: a) resolution-related features, extracted from the image metadata; b) acquisition-related features including repetition time and echo time; c) foreground-related measures including mean values, range and signal-to-noise ratio; d) artifacts-related metrics. The difference in the variance of the extracted measures between training and validation cohorts was tested for significance using Kruskal–Wallis test; p-values below 0.05 were considered statistically significant.

## Results

### Analyzed Volumes

In the MRI data of the training cohort, median volume of the tumoral VOI was 11.7 ml (range: 0.23–121.32 ml) and median volume of the peritumoral VOI was 75.2 ml (range: 7.35–204.53 ml, **Table 1**). In the validation cohort, median volume of the tumoral and peritumoral VOI was 5.0 ml (range: 0.39–48.89 ml) and 53.0 ml (range: 22.19–203.8 ml, **Table 1**), respectively.

### Feature Selection

In the training cohort, the principal component analysis resulted in seven groups of correlated radiomic features for the tumoral VOI (range group size: 15 to 47) and six groups for the peritumoral VOI (range group size: 11 to 62), considering only the images normalized with fixed number of 32 bins. In the linear interpolated images with 50 bin size, six groups for the tumoral VOI (range group size: 23 to 51) and six groups for the peritumoral VOI (range group size: 18 to 49) were built using the principal component analysis.

### Prediction of *MGMT* Promoter Methylation Status

**Figure 2:**
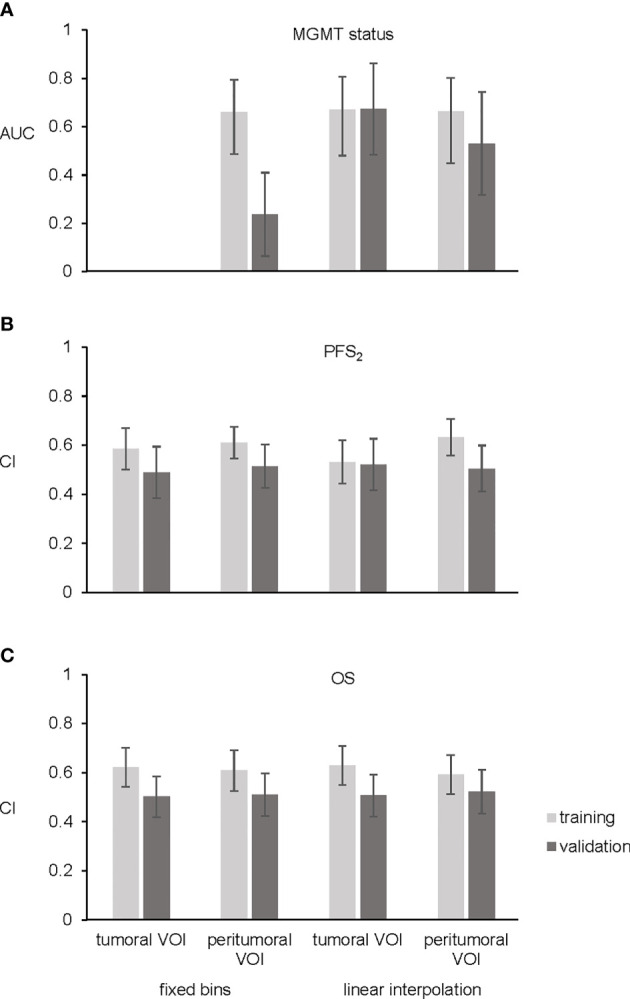
Bar plots. **(A)** Shows area under receiver operating characteristic curve (AUC) of the three final models for *MGMT* status. No significant features were found for prediction of *MGMT* status using tumoral VOI and fixed number of bins. **(B)** Four final models for PFS_2_ showing concordance index (CI). **(C)** CI of the four final OS models. Training cohort in light gray and validation cohort in dark gray. Error bars representing 95% confidence interval.

In the images normalized with fixed number of bins, there was no correlation between radiomic features extracted from the tumoral VOI and *MGMT* status. However, one independent and significant radiomic feature was identified in the peritumoral VOI. The final model (feature: Neighborhood Gray Tone Difference Matrix busyness; AUC 0.660, 95% confidence interval 0.528–0.793, **Figure 2A**) was not validated in the validation cohort (5-fold-cross validation in **Table S3**).

In the images normalized with linear interpolation, three uncorrelated radiomic features from the tumoral VOI and three uncorrelated radiomic features from the peritumoral VOI correlated with *MGMT* status. Upon backward selection of variables, one feature (Neighborhood Gray Level Dependence Matrix low dependence emphasis) in the tumoral VOI and one feature (coefficient of variation) in the peritumoral VOI remained significant. The final model of the tumoral VOI predicting *MGMT* status achieved an AUC of 0.670 (95% CI: 0.5341–0.8056, **Figure 2A** and **Table 2**) and was successfully validated in an independent cohort (AUC 0.673, 95% confidence interval 0.4837–0.8618, **Figure 2A**, 5-fold-cross validation in **Table S3**). In contrast, the peritumoral VOI model showing an AUC of 0.663 (95% CI: 0.5225–0.8024, **Figure 2A**, 5-fold-cross validation in **Table S3**) was not validated.

**Table 2:** Details of the final model for MGMT status prediction.

VOI	feature	coefficient	p-value
tumoral	intercept	−2.46	0.018
tumoral	Neighborhood Gray Level Dependence Matrix low dependence emphasis	5.77	0.036

### Prediction of PFS_2_ and OS

For analysis of radiomics to predict PFS_2_ and OS, MRI data of tumoral and peritumoral VOI were analyzed after intensity normalization using either fixed number of bins or linear interpolation.

#### PFS_2_ Prediction

Multivariable Cox regression in images normalized with fixed number of bins and considering backward selection of variables resulted in two significant radiomic features (Gray Level Size Zone Matrix zone size entropy, Gray Level Size Zone Matrix large zone low gray level emphasis) in the tumoral VOI and two (kurtosis, enhancing tumor volume 70%) in the peritumoral VOI. These analyses resulted in two radiomic models with a CI of 0.585 in the tumoral and a CI of 0.61 in the peritumoral VOI. The multivariable Cox model for both tumoral and peritumoral VOI was not validated (**Figure 2B**, 5-fold-cross validation in **Table S3**). In the training cohort, stratification into two risk groups was significant in the peritumoral VOI (p = 0.02) but not in the tumoral VOI for PFS_2_.

After analyzing data normalized with linear interpolation with multivariable Cox regression based on backward selection of variables, two uncorrelated radiomic features (Gray Level Run Length Matrix long runs emphasis; Gray Level Run Length Matrix run length variance) in the tumoral VOI and two (kurtosis; Grey Level Size Zone Matrix large zone low gray level emphasis) in the peritumoral VOI predicting PFS_2_ remained. These analyses predicting PFS_2_ resulted in two models with a CI of 0.532 in the tumoral VOI and CI of 0.633 in the peritumoral VOI. However, both final models could not be validated in the external validation cohort (**Figure 2B**, 5-fold-cross validation in **Table S3**). In contrast to the images with fixed numbers of bins, the stratification in the training cohort into two risk groups was not significant for tumoral and peritumoral VOI.

#### OS Prediction

In the normalized imaging data with fixed number of bins, two radiomic features (enhancing tumor volume 30%, enhancing tumor volume 40%) for the tumoral VOI and one (enhancing tumor volume 30%) for the peritumoral VOI were entered into the multivariable Cox regression for OS prediction. These analyses resulted in two models with a CI of 0.621 in the tumoral VOI and a CI of 0.609 in the peritumoral VOI. The multivariable Cox model for both, tumoral and peritumoral VOI could not be validated in the external validation cohort (**Figure 2C**, 5-fold-cross validation in **Table S3**). Stratification into two risk groups was not significant.

Multivariable Cox regression of images normalized with linear interpolation led to one radiomic feature (histogram energy) in the tumoral VOI and one (minor axis) in the peritumoral VOI. These analyses resulted in two models with a CI of 0.629 in the tumoral VOI and a CI of 0.592 in the peritumoral VOI, respectively. Both final models could not be validated in the external validation cohort (**Figure 2C**, 5-fold-cross validation in **Table S3**), and stratification into two risk groups was not significant.

### Image Quality

The results of the Kruskal–Wallis test are depicted in **Figure S2**; the investigated metrics and respective p-values are shown in **Table S4**. The test demonstrated a significant difference in variance between cohorts in terms of acquisition protocols: both variances in repetition time and echo time in the training cohort are significantly larger from those in the validation one, p-values of 0.027 and 0.005 for repetition time and echo time, respectively. In the MRI data of our training cohort, slice thickness varied from 0.44 to 6.6 mm (median, 1 mm) and in-plane resolution varied from 0.36 to 1.2 mm, whereas in the validation cohort slice thickness varied from 0.7 to 5.3 mm (median, 1.5 mm) and in-plane resolution from 0.38 to 0.99 mm. Only the variance of y voxel dimension was significantly larger in the training cohort. Additionally, variance of the entropy focus criterion, which describes motion artifacts, was significantly larger (p = 0.013) in the training cohort. Unexpectedly, despite the fact that about 71% of the validation cohort was collected in a single institution, substantial variation in the image quality was observed, which corresponds to the variation observed (**Figure S2**) in the multicenter data collection (training cohort).

## Discussion

In this study, we performed a radiomic analysis on gadolinium contrast enhanced T1-weighted MR images from patients with recurrent glioblastoma after alkylating chemotherapy. In terms of analyzed volume of interest, two volumes, tumoral and peritumoral, were included. Data for the training cohort are based on the prospective randomized DIRECTOR trial, which showed similar outcome in both arms (9). Validation data were obtained from a matched in-house recurrent glioblastoma patient cohort (27, 28).

The proposed radiomic model reliably predicted *MGMT* status from MRI contrast enhancing tumor regions after intensity normalization with linear interpolation in an independent cohort. This result has been reported previously in a radiomic analysis only from patients with newly diagnosed gliomas prior to standardized treatment (16, 29–33). In contrast, our models for *MGMT* prediction failed for intensity normalization with fixed bins or radiomic data from the peritumoral region. This finding highlights the importance of intensity normalization in quantitative MR analysis. Two models predicting PFS_2_ and OS, respectively, were trained. However, neither of these models could be validated in the independent external patient cohort, and differences in models’ performances were not statistically significant. Despite the ability to build prognostic models for outcome in the DIRECTOR cohort, the model ultimately failed in an external cohort, emphasizing the need for independent validation of model results for generalized applicability as mandated by the TRIPOD statement (34).

The key strengths of our study are the heterogeneity of the imaging data with regard to scanner models and image acquisition protocols in our training cohort collected during a prospective multicenter study (9) as well as the normalization methods. We hypothesized that finding a model in such a heterogeneous data pool would improve validation success in an independent cohort. However, variation is not only restricted to patients treated in different centers but also extends to different MRI acquisition protocols within one center, see **Figure S2** (*e.g.* different scanners, magnetic field strength, MRI slice thickness, or in plane resolution). Therefore, we performed voxel size resampling to a common resolution of 3 mm. The advantage of this method is the comparability of images, which is commonly used in radiomic research (19). On the other hand, much information from the images with originally small cubic voxel sizes is lost. In addition, artificial data is introduced for images with originally large vox sizes. Whereas voxel size interpolation is standard in radiomic analysis, intensity normalization is often omitted (35, 36). We tested two different intensity normalization techniques to standardize grayscale MRI data to reduce interpatient and interstudy variability of the images. The simpler methods, fixed number of bins normalization, causes loss of information about absolute range of observed intensities. Using this method, none of the models could be validated. The second linear intensity interpolation is more labor intensive, as it requires segmentation of two additional structures. However, this method has the advantage of preserving information about absolute intensity range within the tumor. Importantly, this method allowed for successful validation of the *MGMT* status model, improving comparability between cohorts, while preserving information about tumor biology. The PFS_2_ and OS models, however, could not be validated. This might be explained by much higher complexity of the endpoint and non-standardized treatment. On the technical side, the results might be further improved with application of bias field correction (37). Although the method applied for feature selection results in a restrictive and small number of features, as it has been shown to be superior to alternative approaches: recent findings show the method used here delivers the best models in comparison to maximum relevance minimum redundancy, mutual information and least absolute shrinkage and selection operator (LASSO) methods (38).

Model building, validation and reporting should be performed according to the TRIPOD statement which distinguishes different levels corresponding to the model validation process (34). Most studies using radiomic approaches in glioblastoma correspond to type 1b, 2a, or 2b analyses, where the model is trained and only validated on the data from the same or similar origin (see reviews by Park and colleagues) (39). When relying only on internal validation data, such models risk of overfitting and may provide an optimistic estimate of prediction performance. In addition, for a model to be used as a broadly applicable decision-making tool, external validation is mandatory. Park and colleagues reported that 63 out of 77 radiomic studies lack external validation (81.8%) (39). To overcome this limitation, we aimed to perform a TRIPOD statement type 3 model development using an independent curated patient cohort for validation. So far, only few studies have reported their model results based on this validation type (30, 40, 41). In the final model evaluation on an external dataset, we were not able to validate our models for PFS_2_ and OS, but for *MGMT* prediction. Therefore, we consider our results as reliable and robust and conclude that the use of TRIPOD level 3 should be a prerequisite for a model’s applicability in routine clinical use.

A possible explanation for the failure to validate the models for outcome prediction may be due to biological aspects of glioblastoma progression and alterations due to treatment. Draaisma and colleagues (42) showed that tumor biology differs at the genetic level from first presentation to recurrence, suggesting alterations in tumor biology over the course of disease. The primary treatment (*e.g.* different amounts of scaring tissue, resection cavities due to surgery) may also influence the presentation of recurrent tumor on MR images. Additionally, tumor volume at recurrence is often very small, thus yielding less tumor information for calculations (*e.g.* wavelet transformation features) compared to MRIs obtained at initial diagnosis. Furthermore, reproducibility of the contours is limited due to an unclear distinction between cystic or necrotic areas and resection cavities. Contouring could be improved, however, by comparison to MRI after first resection. Another limitation of our model is the small sample size available for this analysis, thus indicating the need of further investigations in a larger cohort. Finally, incorporating additional sequences such as T2-weighted and FLAIR gaining complementary radiomic information may improve prediction.

Even though the current results are promising, the vast majority of the studies in the growing field of quantitative radiomics have analyzed newly diagnosed glioblastomas. Only a few radiomic approaches have been published on glioblastomas at recurrence (15, 43, 44). Since our study focuses on recurrent glioblastoma, we provide additional models for predictive and diagnostic criteria for patients with a poor prognosis. This finding may represent a small but significant step towards highlighting the clinical relevance of radiomic approaches for newly diagnosed glioblastoma.

In conclusion, our model predicts *MGMT* promoter methylation status based on tumor texture features on gadolinium-enhanced T1-weighted MRI in patients with recurrent glioblastoma treated with alkylating chemotherapy. Therefore, our model provides a non-invasive approach to predict patient response to chemotherapy. However, the radiomic approach to predict PSF_2_ and OS remained unsuccessful for patients with recurrent glioblastoma.

## Data Availability Statement

The datasets presented in this article are not readily available because patient individual data has been taken from a prospective trial which would need consent of all involved study PI if data sharing upon request is initiated. Requests to access the datasets should be directed to nicolaus.andratschke@usz.ch.

## Ethics Statement

The studies involving human participants were reviewed and approved by the local ethics committees and designated authorities (KEK-ZH-Nr. 20140540, KEK-ZH-Nr. 2009-0135/1, KEK-ZH-Nr. 2015-0437). The patients/participants provided their written informed consent to participate in this study.

## Author Contributions

Study concept and design: AV, MB, ST-L, MG, NA. Collection and provision of patient data: H-GW, DG, GR, ER, WW, MW. Performance of data processing and analysis: AV, MB, ST-L, DV, NS, JK, H-GW, DG, NA. Data interpretation and postprocessing: AV, MB, ST-L, NA. Writing the manuscript: AV, MB, NS, NA. Revision of the manuscript for important intellectual content: AV, MB, ST-L, DV, NS, JK, H-GW, DG, WW, ER, GR, MG, MW, NA. All authors contributed to the article and approved the submitted version.

## Funding

The research project has been partially funded by the Clinical Research Priority Program “Artificial Intelligence in Oncological Imaging” of the University of Zurich. The research project was partially funded by SPHN IMAGINE.

## Conflict of Interest

The authors declare that the research was conducted in the absence of any commercial or financial relationships that could be construed as a potential conflict of interest.

## Abbreviations

AUC, area under receiver operating characteristic curve; CI, concordance index; *MGMT* status, O6-methylguanine DNA methyltransferase promoter methylation status; OS, overall survival; PFS_2,_ progression free survival; VOI, volume of interest.
