# Supplementary material for: Radiomic Analysis to Predict Outcome in Recurrent Glioblastoma Based on Multi-Center MR Imaging From the Prospective DIRECTOR Trial
Source: Front Oncol. 2021 Apr 14;11:636672. doi: 10.3389/fonc.2021.636672 (PMC8079773; doi:10.3389/fonc.2021.636672)
Supplement: Supplementary file 1 [file DataSheet_1.docx]

Supplementary Material

# Supplementary Figure and Tables

## Supplementary Figure


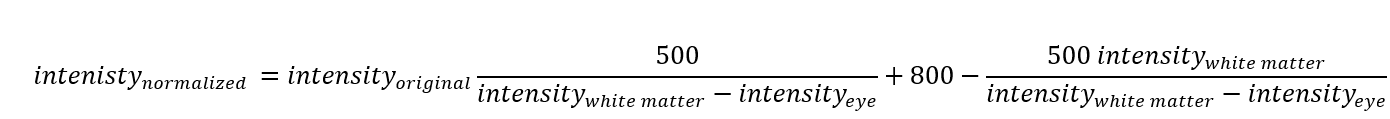


**Supplementary Figure 1**. Function showing the relation of the original to the transformed intensities assuming that standard intensity eye = 300 and standard intensity-white matter = 800.


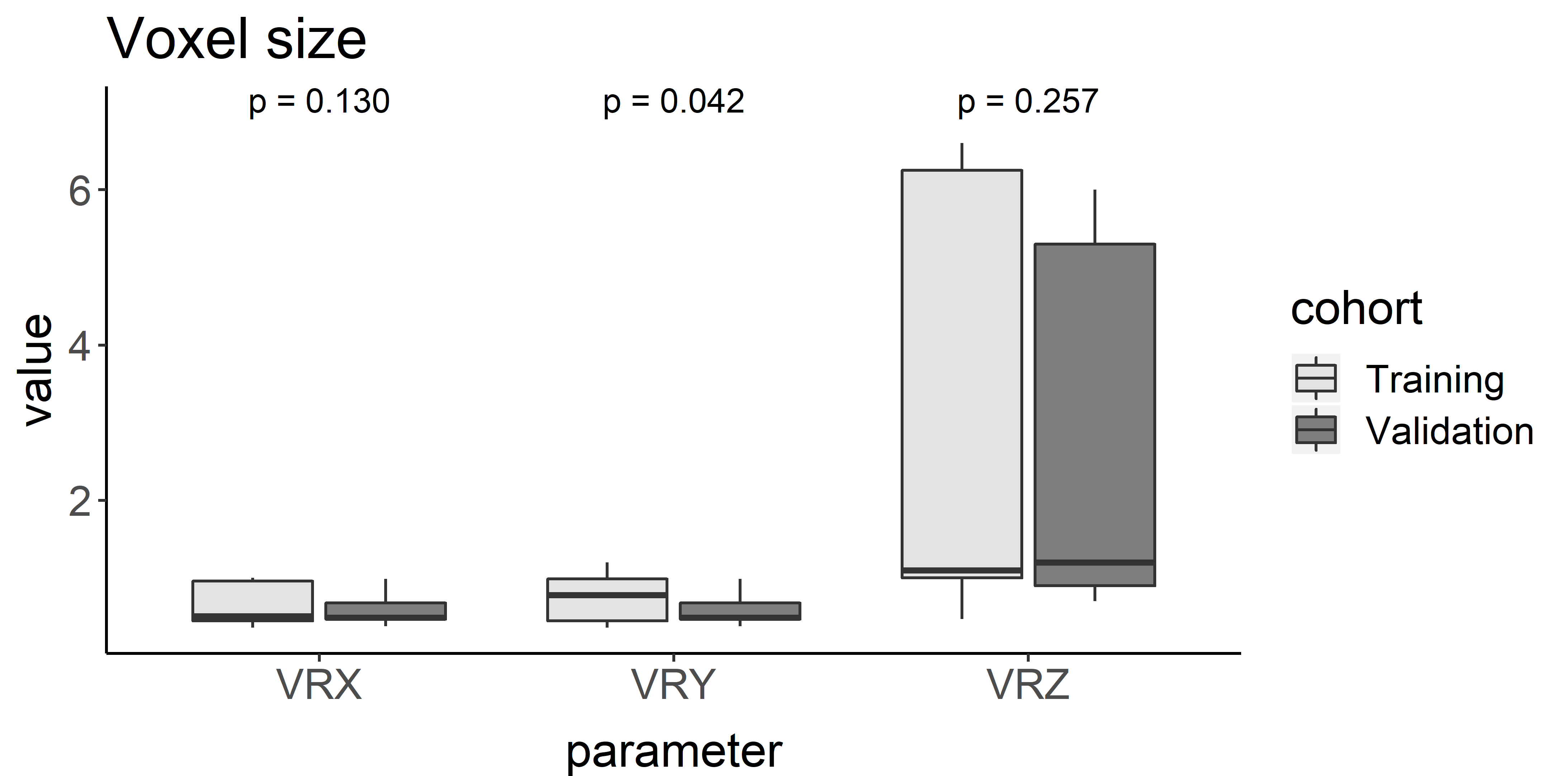


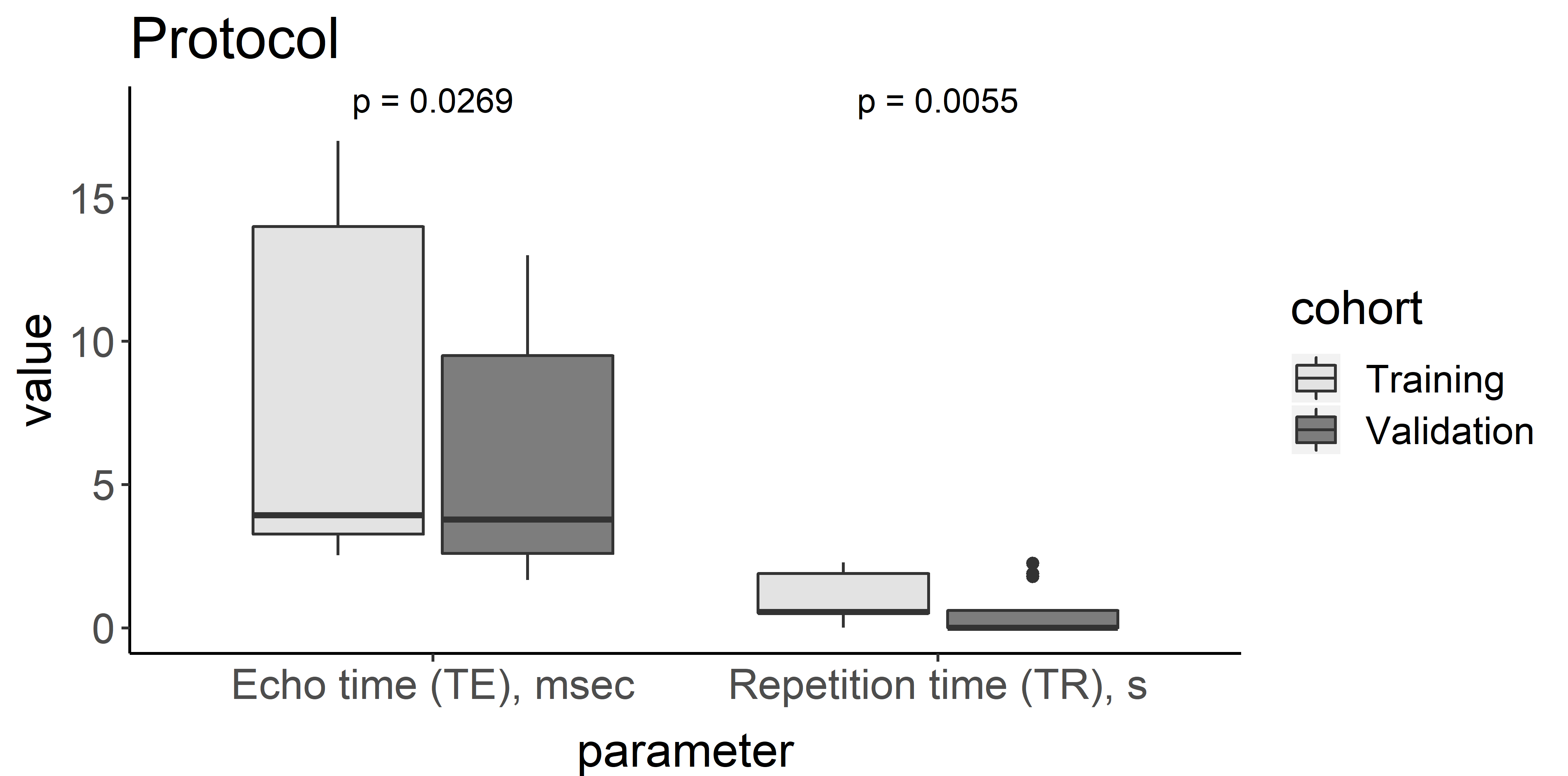


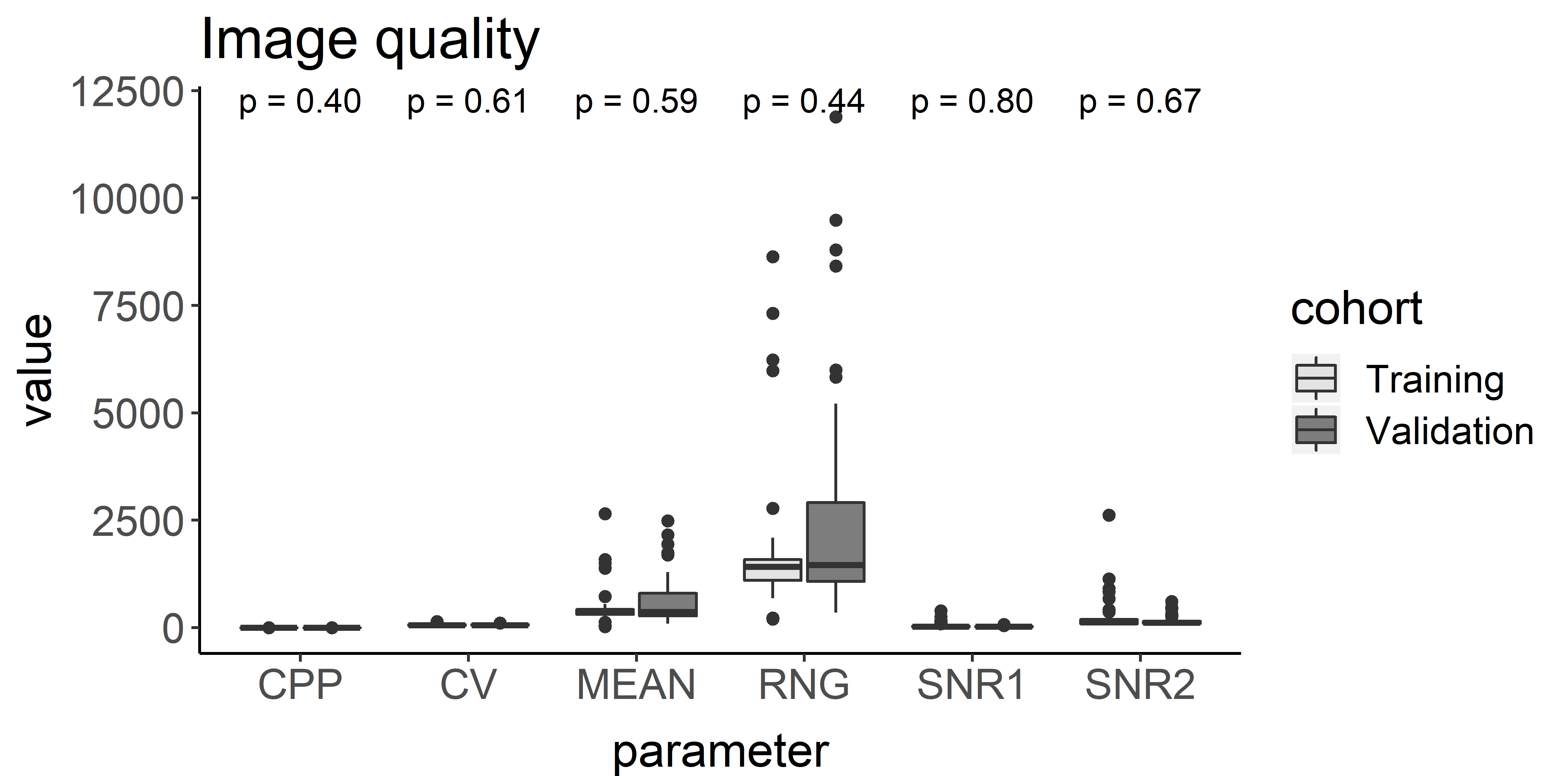


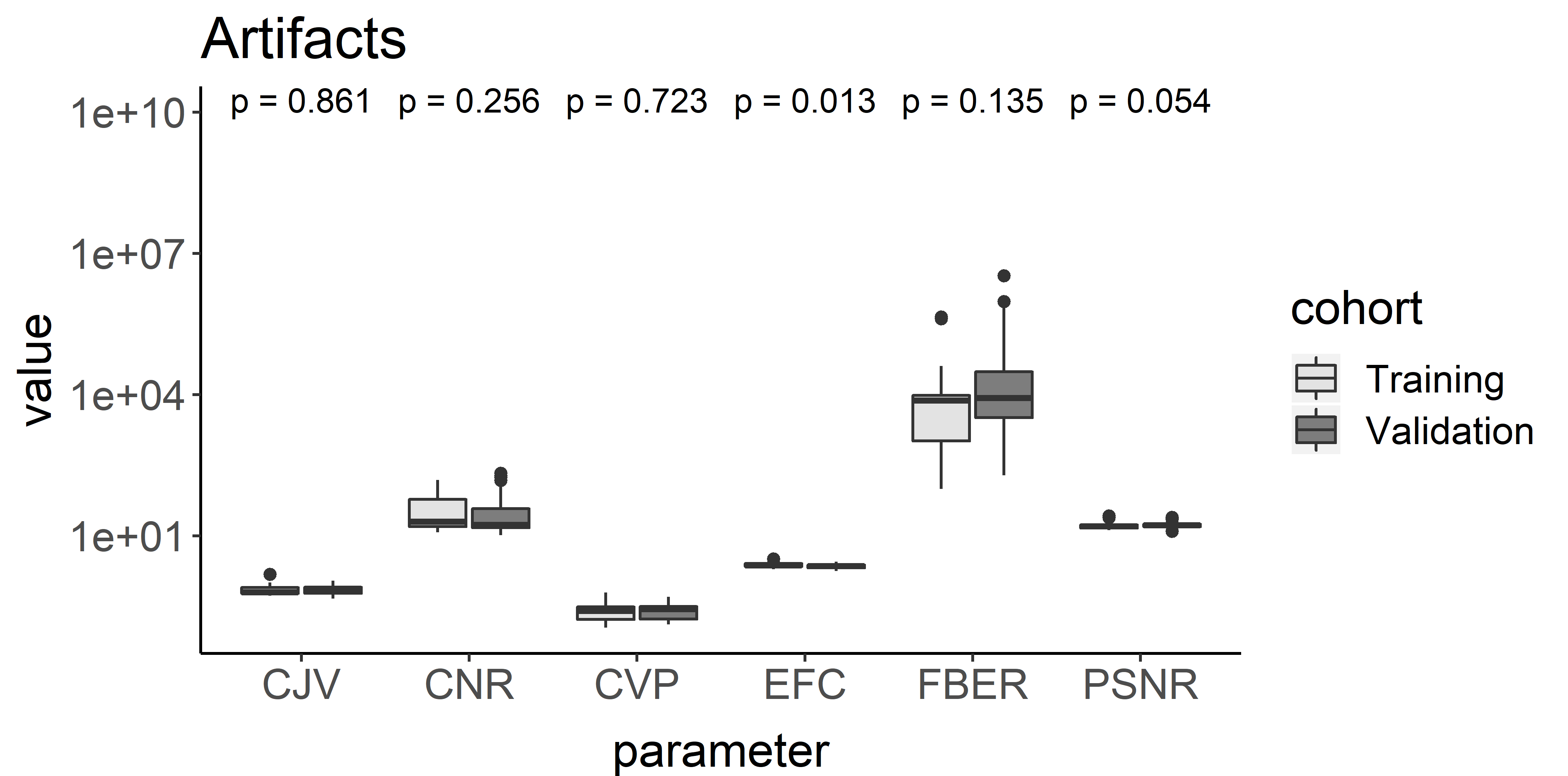


**Supplementary Figure 2.** Difference in variance of the metadata and quality measures between training and validation cohorts tested for significance with Kruskal-Wallis test and the respective p-values.

## Supplementary Tables

| **DIRECTOR trial inclusion criteria** | **Additional exclusion criteria for present study** |
| --- | --- |
| - progressive or recurrent glioblastoma - progressive or recurrent glioblastoma - tissue available for the determination of MGMT status - prior treatment with TMZ/RT - informed consent - age 18 to 80 years - Karnofsky performance score ≥ 50% - absolute neutrophil counts > 1,500/mL; platelet counts > 100,000/mL; hemoglobin > 10 g/dL; - serum creatinin < 1.5-fold upper normal range - ASAT or ALAT < 3-fold upper normal range unless attributed to anticonvulsants - alkaline phosphatase < 3-fold upper normal range - women with childbearing potential must have a negative serum pregnancy | - unavailable pre- and post-contrast T1-weighted MR imaging data at recurrence prior to second surgery - slice thickness of imaging data larger than 6.6 mm - tumor volume at recurrence smaller 73 than 0.2 ml - newly diagnosed tumor located in the spinal cord |

**Supplementary Table 1.** Inclusion and exclusion criteria.

| radiomics type | matrix | feature name | IBSI reference |
| --- | --- | --- | --- |
| shape | shape | volume |  |
|  |  | surface |  |
|  |  | compactness_1 |  |
|  |  | compactness_2 |  |
|  |  | spherical disproportion |  |
|  |  | sphericity |  |
|  |  | asphericity |  |
|  |  | surface to volume ratio |  |
|  |  | median thickness | Feature not defined in IBSI, see(1) |
|  |  | standard deviation thickness | Feature not defined in IBSI, see(1) |
|  |  | euclidian distance | Feature not defined in IBSI, see(1) |
|  |  | major axis length |  |
|  |  | minor axis length |  |
|  |  | least axis length |  |
|  |  | elongation |  |
|  |  | flatness |  |
|  |  | fractal dimension | Feature not defined in IBSI, see(1) |
|  |  | center of mass shift |  |
|  |  | cumulative volume 20% | Features not specified in IBSI. They refer to a region of interest volume above given threshold T (e.g. 20%) of maximum intensity in this region. Analogous to metabolic tumor volume in PET imaging |
|  |  | cumulative volume 30% |  |
|  |  | cumulative volume 40% |  |
|  |  | cumulative volume 50% |  |
|  |  | cumulative volume 60% |  |
|  |  | cumulative volume 70% |  |
| intensity | histogram | minimum |  |
|  |  | maximum |  |
|  |  | mean |  |
|  |  | standard deviation |  |
|  |  | coefficient of variation |  |
|  |  | skewness |  |
|  |  | kurtosis |  |
|  |  | variance |  |
|  |  | median |  |
|  |  | percentile 10th |  |
|  |  | percentile 90th |  |
|  |  | interquartile range |  |
|  |  | range |  |
|  |  | mean absolute deviation |  |
|  |  | robust mean absolute deviation |  |
|  |  | energy |  |
|  |  | entropy |  |
|  |  | root mean square |  |
|  |  | uniformity |  |
| texture | GLCM | energy |  |
|  |  | entropy |  |
|  |  | contrast |  |
|  |  | correlation |  |
|  |  | homogeneity |  |
|  |  | homogeneity normalized |  |
|  |  | inverse difference |  |
|  |  | inverse difference normalized |  |
|  |  | variance |  |
|  |  | sum of average |  |
|  |  | sum of entropy |  |
|  |  | sum of variance |  |
|  |  | difference entropy |  |
|  |  | difference variance |  |
|  |  | information measures of correlation 1 |  |
|  |  | information measures of correlation 2 |  |
|  |  | maximal correlation coefficient | Feature not defined in IBSI, see(1) |
|  |  | joint maximum |  |
|  |  | joint average |  |
|  |  | difference average |  |
|  |  | dissimilarity |  |
|  |  | inverse variance |  |
|  |  | autocorrelation |  |
|  |  | cluster tendency |  |
|  |  | cluster shade |  |
|  |  | cluster prominence |  |
|  | merged  GLCM | energy |  |
|  |  | entropy |  |
|  |  | contrast |  |
|  |  | correlation |  |
|  |  | homogeneity |  |
|  |  | homogeneity normalized |  |
|  |  | inverse difference |  |
|  |  | inverse difference normalized |  |
|  |  | variance |  |
|  |  | sum of average |  |
|  |  | sum of entropy |  |
|  |  | sum of variance |  |
|  |  | difference entropy |  |
|  |  | difference variance |  |
|  |  | information measures of correlation 1 |  |
|  |  | information measures of correlation 2 |  |
|  |  | maximal correlation coefficient | Feature not defined in IBSI, see(1) |
|  |  | joint maximum |  |
|  |  | joint average |  |
|  |  | difference average |  |
|  |  | dissimilarity |  |
|  |  | inverse variance |  |
|  |  | autocorrelation |  |
|  |  | cluster tendency |  |
|  |  | cluster shade |  |
|  |  | cluster prominence |  |
|  | NGTDM | coarseness |  |
|  |  | contrast |  |
|  |  | busyness |  |
|  |  | complexity |  |
|  |  | strength |  |
|  | GLRLM | gray-level non-uniformity |  |
|  |  | gray-level non-uniformity normalized |  |
|  |  | run length non-uniformity |  |
|  |  | run length non-uniformity normalized |  |
|  |  | short run emphasis |  |
|  |  | long runs emphasis |  |
|  |  | low gray-level run emphasis |  |
|  |  | high gray-level run emphasis |  |
|  |  | short run low gray-level emphasis |  |
|  |  | short run high gray-level emphasis |  |
|  |  | long run low gray-level emphasis |  |
|  |  | long run high gray-level emphasis |  |
|  |  | run percentage |  |
|  |  | gray level variance |  |
|  |  | run length variance |  |
|  |  | run entropy |  |
|  | merged  GLRLM | gray-level non-uniformity |  |
|  |  | gray-level non-uniformity normalized |  |
|  |  | run length non-uniformity |  |
|  |  | run length non-uniformity normalized |  |
|  |  | short run emphasis |  |
|  |  | long runs emphasis |  |
|  |  | low gray-level run emphasis |  |
|  |  | high gray-level run emphasis |  |
|  |  | short run low gray-level emphasis |  |
|  |  | short run high gray-level emphasis |  |
|  |  | long run low gray-level emphasis |  |
|  |  | long run high gray-level emphasis |  |
|  |  | run percentage |  |
|  |  | gray level variance |  |
|  |  | run length variance |  |
|  |  | run entropy |  |
|  | GLSZM | gray-level non-uniformity |  |
|  |  | gray-level non-uniformity normalized |  |
|  |  | size zone non-uniformity |  |
|  |  | size zone non-uniformity normalized |  |
|  |  | small zone emphasis |  |
|  |  | large zone emphasis |  |
|  |  | low gray-level zone emphasis |  |
|  |  | high gray-level zone emphasis |  |
|  |  | small zone low gray-level emphasis |  |
|  |  | small zone high gray-level emphasis |  |
|  |  | large zone low gray-level emphasis |  |
|  |  | large zone high gray-level emphasis |  |
|  |  | zone percentage |  |
|  |  | gray level variance |  |
|  |  | zone size variance |  |
|  |  | zone size entropy |  |
|  | GLDZM | gray-level non-uniformity | Definition of distance differs from IBSI, see(1) |
|  |  | gray-level non-uniformity normalized |  |
|  |  | zone distance non-uniformity |  |
|  |  | zone distance non-uniformity normalized |  |
|  |  | small distance emphasis |  |
|  |  | large distance emphasis |  |
|  |  | low gray-level zone emphasis |  |
|  |  | high gray-level zone emphasis |  |
|  |  | small distance low gray-level emphasis |  |
|  |  | small distance high gray-level emphasis |  |
|  |  | large distance low gray-level emphasis |  |
|  |  | large distance high gray-level emphasis |  |
|  |  | zone percentage |  |
|  |  | gray level variance |  |
|  |  | zone distance variance |  |
|  |  | zone distance entropy |  |
|  | NGLDM | gray-level non-uniformity |  |
|  |  | gray-level non-uniformity normalized |  |
|  |  | dependence count non-uniformity |  |
|  |  | dependence count non-uniformity normalized |  |
|  |  | low dependence emphasis |  |
|  |  | high dependence emphasis |  |
|  |  | low gray-level count emphasis |  |
|  |  | high gray-level count emphasis |  |
|  |  | low dependence low gray-level emphasis |  |
|  |  | low dependence high gray-level emphasis |  |
|  |  | high dependence low gray-level emphasis |  |
|  |  | high dependence high gray-level emphasis |  |
|  |  | gray level variance |  |
|  |  | dependence count variance |  |
|  |  | dependence count entropy |  |
|  |  | dependence count energy |  |

**Supplementary Table 2.** Radiomic features. Full list of radiomic features used in this study is presented below. Z-Rad calculates radiomic features (intensity and texture) according to the image biomarker standardization initiative (IBSI, version 9(1, 2)). Features in the Tab S3 are referenced to appropriate paragraph in IBSI documentation or if feature is not implemented in IBSI details of the calculation are specified. GLCM – gray level co-occurrence matrix, NGTDM – neighborhood gray tone difference matrix, GLRLM – gray level run length matrix, GLSZM – gray level size zone matrix, GLDZM – gray level distance zone matrix, NGLDM – neighboring gray level dependence matrix.

|  | **Fixed bins** | | | | | |
| --- | --- | --- | --- | --- | --- | --- |
|  | tumoral VOI | | | peritumoral VOI | | |
|  | OS | PFS | MGMT | OS | PFS | MGMT |
| Mean | 0.591 | 0.582 | - | 0.591 | 0.601 | 0.77 |
| Range | 0.522-0.718 | 0.512-0.640 | - | 0.444-0.798 | 0.473-0.708 | 0.667-0.889 |
|  | **Linear interpolation** | | | | | |
|  | tumoral VOI | | | peritumoral VOI | | |
|  | OS | PFS | MGMT | OS | PFS | MGMT |
| Mean | 0.661 | 0.493 | 0.653 | 0.591 | 0.657 | 0.706 |
| Range | 0.570-0.769 | 0.373-0.603 | 0.425-0.778 | 0.494-0.697 | 0.551-0.731 | 0.551-0.889 |

**Supplementary Table 3.** Internal 5-fold cross validation.

| **Parameter** | **Abbreviation** | **Kruskal-Wallis, p-value** |
| --- | --- | --- |
| Coefficient of joint variation between the foreground and background | CJV | 0.861 |
| Contrast to noise ratio | CNR | 0.256 |
| Columns value | COLS | 0.007 |
| Contrast per pixel | CPP | 0.397 |
| Coefficient of variation | CV | 0.611 |
| Coefficient of variation of the foreground patch (for shading artifacts) | CVP | 0.723 |
| Entropy focus criterion (for motion artifacts) | EFC | 0.013 |
| Foreground-background energy ratio (for ringing artifacts) | FBER | 0.159 |
| Mean of the foreground | MEAN | 0.594 |
| Number of slice images i | NUM | 0.590 |
| Peak signal to noise ratio of the foreground | PSNR | 0.054 |
| Range of the foreground | RNG | 0.442 |
| Raws value | ROWS | 0.015 |
| Foreground standard deviation (SD) divided by background | SNR1 | 0.798 |
| Mean of the foreground patch divided by background | SNR2 | 0.669 |
| Foreground patch SD divided by the centered foreground patch | SNR3 | 0.887 |
| Mean of the foreground patch divided by mean of the background patch | SNR4 | 0.266 |
| Echo time | TE | 0.027 |
| Repetition time | TR | 0.005 |
| Variance of the foreground | VAR | 0.497 |
| Voxel resolution in x plane | VRX | 0.130 |
| Voxel resolution in y plane | VRY | 0.042 |
| Voxel resolution in z plane | VRZ | 0.257 |

**Supplementary Table 4.** Metadata and quality measures extracted within MRQy.

# References

1. USZ Medical Physics [cited 2020 13 Feb]. Available from: <https://medical-physics-usz.github.io/>.

2. Zwanenburg A, Vallières M, Abdalah MA, Aerts H, Andrearczyk V, Apte A, et al. The Image Biomarker Standardization Initiative: Standardized Quantitative Radiomics for High-Throughput Image-based Phenotyping. *Radiology* (2020) 295(2):328-38. Epub 2020/03/11. doi: 10.1148/radiol.2020191145. PubMed PMID: 32154773; PubMed Central PMCID: PMCPMC7193906.
